# Comparison of Various Chromatographic Systems for Identification of Vortioxetine in Bulk Drug Substance, Human Serum, Saliva, and Urine Samples by HPLC-DAD and LC-QTOF-MS

**DOI:** 10.3390/molecules25112483

**Published:** 2020-05-27

**Authors:** Anna Petruczynik, Karol Wróblewski, Krzysztof Wojtanowski, Tomasz Mroczek, Dariusz Juchnowicz, Hanna Karakuła-Juchnowicz, Tomasz Tuzimski

**Affiliations:** 1 Department of Inorganic Chemistry, Medical University of Lublin, Chodźki 4a, 20-093 Lublin, Poland; 2 Department of Experimental and Clinical Pharmacology, University of Rzeszów, Kopisto 2a, 35-959 Rzeszów, Poland; 3 Laboratory for Innovative Research in Pharmacology, University of Rzeszów, Kopisto 2a, 35-959 Rzeszów, Poland; 4 Department of Pharmacognosy with Medicinal Plant Unit, Medical University of Lublin, Chodźki 1, 20-093 Lublin, Poland; krzysztof.wojtanowski@umlub.pl (K.W.); tomasz.mroczek@umlub.pl (T.M.); 5 Department of Psychiatric Nursing, Medical University of Lublin, Chodźki 7, 20-124 Lublin, Poland; dariusz.juchnowicz@umlub.pl; 6 1st Department of Psychiatry, Psychotherapy and Early Intervention, Medical University of Lublin, Głuska 1, 20-439 Lublin, Poland; hanna.karakula-juchnowicz@umlub.pl; 7 Department of Clinical Neuropsychiatry, Medical University of Lublin, 20-439 Lublin, Poland; 8 Department of Physical Chemistry, Medical University of Lublin, Chodźki 4a, 20-093 Lublin, Poland

**Keywords:** vortioxetine, HPLC-DAD, HPLC-QTOF-MS, SPE, saliva, serum, urine, lipophilicity

## Abstract

*Background*: Determination of psychotropic drugs in clinical study is significant, and the establishment of methodologies for these drugs in biological matrices is essential for patients’ safety. The search for new methods for their detection is one of the most important challenges of modern scientific research. The methods for analyzing of psychotropic drugs and their metabolites in different biological samples should be based on combining a very efficient separation technique including high-performance liquid chromatography (HPLC), with a sensitive detection method and effectively sample preparation methods. *Objective*: Retention, peaks symmetry and system efficiency of vortioxetine on Hydro RP, Polar RP, HILIC A (with silica stationary phase), HILIC-B (with aminopropyl stationary phase), and ACE HILIC-N (with polyhydroxy stationary phase and SCX columns were investigated. Various mobile phases containing methanol or acetonitrile as organic modifiers and different additives were also applied to obtained optimal retention, peaks shape, and systems efficiency. The best chromatographic procedure was used for simultaneous analysis of vortioxetine and its metabolites in human serum, urine and saliva samples. *Methods*: Analysis of vortioxetine was performed in various chromatographic systems: Reversed phase (RP) systems on alkylbonded or phenyl stationary phases, hydrophilic interaction liquid chromatography (HILIC), and ion-exchange chromatography (IEC). Based on the dependence of log *k* vs the concentration of the organic modifier, log *kw* values for vortioxetine in various chromatographic systems were determined and compared with calculated log *P* values. Solid phase extraction (SPE) method was applied for sample pre-treatment before HPLC analysis. HPLC-QTOF-MS method was applied for confirmation of presence of vortioxetine and some its metabolites in biological samples collected from psychiatric patient. *Conclusions*: Differences were observed in retention parameters with a change of the applied chromatographic system. The various properties of stationary phases resulted in differences in vortioxetine retention, systems’ efficiency, and peaks’ shape. Lipophilicity parameters were also determined using different HPLC conditions. The most optimal systems were chosen for the analysis of vortioxetine in biological samples. Both serum and urine or saliva samples collected from patients treated with vortioxetine can be used for the drug determination. For the first time, vortioxetine was detected in patient’s saliva. Obtained results indicate on possibility of application of saliva samples, which collection are non-invasive and painless, for determination and therapeutic drug monitoring in patients.

## 1. Introduction

Major depressive disorder is a main cause of disability and is associated with significant reductions in quality of life, have a negative effect on productivity, reduced overall health and significant economic costs. The most frequently used pharmacological treatments for major depressive disorder include the selective serotonin reuptake inhibitors and the serotonin and norepinephrine reuptake inhibitors [1]. However, these drugs can may generate only a limited antidepressant response, improved responses have been obtained with different or combined therapies involving additional mechanisms, such as the allosteric serotonin reuptake inhibitor escitalopram or by combinations of medications.

Vortioxetine (1-[2-(2,4-dimethylphenylsulfanyl)-phenyl]-piperazine hydrobromide is an antidepressant (approved by the U.S. FDA for the treatment of major depressive disorder in 2013) with a multi-modal mechanism of action approved for the treatment of major depressive disorder. The drug is 5-HT3-R, 5-HT7-R, and 5-HT1D-R antagonist, 5-HT1B-R partial agonist, 5-HT1A-R agonist, and serotonin (5-HT) transporter inhibitor [2]. Additionally, vortioxetine shows pro-cognitive activity in animal models and was observed beneficial effects of vortioxetine treatment on cognitive dysfunction in major depressive patients [3].

A literature review revealed that only few liquid chromatography (LC) methods have been reported for vortioxetine analysis. For analysis of vortioxetine in various samples different LC procedures were applied. Currently, reversed phase liquid chromatography (RP LC) is considered to be the gold standard in pharmaceutical analysis, and success of the method is attributed to the fact that this chromatographic mode matches perfectly with the physicochemical properties of drugs [4]. Most HPLC analysis were performed on C18 [5,6,7,8,9,10,11] or C8 [12] columns with aqueous mobile phases containing acetonitrile [5,6,7,8,9,11,12] or rarely methanol [10] as organic modifiers. The addition of acids (most often formic acid [5,6,7,11,12], rarely trifluoroacetic acid [9]) and silanol blockers [8,10] to aqueous-organic mobile phases were usually applied for vortioxetine determination. Ion-exchange chromatography was also rarely used for analysis of vortioxetine [13]. 

For the analysis of vortioxetine, liquid chromatography combined with mass spectrometry (LC-MS) or tandem mass spectrometry (LC-MS/MS) was most commonly used. Ultra performance liquid chromatography with tandem mass spectrometry (UPLC–MS/MS) was used for simultaneous determination of the concentrations of vortioxetine, carvedilol and its active metabolite 4-hydroxyphenyl carvedilol in rat plasma [5]. Analysis was performed on C18 column with mobile phase containing acetonitrile, water and formic acid. Triple quadrupole mass spectrometer equipped with an electro-spray ionization (ESI) source was applied for detection. Similar UPLC-MS/MS procedure was also applied by Gu et al. to the pharmacokinetic study of vortioxetine in rat plasma after oral administration [6]. Analysis of vortioxetine in various biological samples (rat plasma, urine, feces) for pharmacokinetic study was performed by UHPLC-MS/MS method on C18 column [7]. Mobile phase containing acetonitrile, water, and formic acid was also applied in the investigations. LC-DAD and LC-MS methods were also applied for determination of the concentrations of vortioxetine in human serum and saliva samples [10]. In the method, phenyl stationary phase (Polar RP column) and the mobile phase containing methanol, acetate buffer at pH 3.5 and diethylamine were applied for LC-DAD and LC-MS analysis. Vortioxetine and fluoxetine were analyzed in rat brains by the similar chromatographic system on C18 column with mobile phase containing acetonitrile, water, and formic acid [11]. A simultaneously analysis of deuvortioxetine, vortioxetine and their carboxylic acid metabolite in rat plasma was performed by LC-MS/MS method [13]. Chromatographic separation was performed on C18 column with mobile phase containing methanol, formic acid and ammonium formate. The method was applied to a toxicokinetic study. Vortioxetine was also determined by LC-MS/MS in blood plasma and brain homogenates of mice [14]. Analysis were performed on Accucore RP-MS column. Mobile phase consisted of methanol, formic acid and ammonium formate. 

Vortioxetine was sometimes analyzed in pharmaceutical formulation. De Diego et al. determined vortioxetine and its degradation product in bulk and tablets by liquid chromatography with diode array detector (LC-DAD) and LC-MS/MS using C18 column and mixture containing acetonitrile, water, acetic acid, and trimethylamine as mobile phase [8]. Liu et al. developed and validated of a stability-indicating RP HPLC method for the separation and identification of potential impurities in vortioxetine [9]. After optimization of HPLC method analysis of vortioxetine and impurities was performed on C18 column with mobile phase consisting of acetonitrile, water, and trifluoroacetic acid. For confirmation of presence of vortioxetine impurities nuclear magnetic resonance (NMR), MS, and infrared spectroscopy (IR) were applied.

There have been few published procedures for the determination of vortioxetine in samples obtained from patients treated with the drug. Chen et al. investigated the potential pharmacokinetic interactions between vortioxetine and co-administered fluconazole, ketoconazole, rifampicin, bupropion, ethinyl estradiol/levonorgestrel, and omeprazole for avoiding or minimizing the interaction-induced adverse events associated with specific drug combinations [13]. They determined the concentration of vortioxetine in human plasma by HPLC-MS/MS on ion-exchange column using mobile phase containing acetonitrile, water, and ammonium formate.

The aim of this study was comparison of various chromatographic systems for analysis of vortioxetine in bulk drug substance, serum, saliva, and urine by HPLC with diode array (DAD) and mass spectrometry detection (QTOF-MS). Reversed phase (RP), HILIC and ion-exchange systems were examined for this purpose. The most efficient and selective systems were chosen for analysis of vortioxetine in biological samples. HPLC was also applied for determination of vorioxetine lipophilicity in various chromatographic systems. The obtained lipofilicity parameters were compared with log *P* values calculated by computer programs. For the first time, detection of vortioxetine in human saliva from patient treated by this drug was performed.

## 2. Results and Discussion

### 2.1. Comparison of Chromatographic Systems

Vortioxetine standard was chromatographed on different columns in various eluent systems for choose the optimal chromatographic systems for analysis of the compound in biological samples. The analyses were performed using RP, ion exchange, and HILIC modes. Tested chromatographic systems were compared in terms of retention of vortioxetine, peak shape and system efficiency (Table 1). 

#### 2.1.1. Analysis of Vortioxetine on Octadecyl Stationary Phase

In the first step of investigations commonly used octadecyl (C18) stationary phase with various mobile phases was applied. The dependence of vortioxetine retention vs the concentration of acetonitrile or methanol in the mobile phase containing organic modifier, water and 0.1% formic acid was examined. In system containing acetonitrile in the whole concentration range peaks obtained for investigated drug were very asymmetrical (Table S1). Only in system with mobile phase containing 30% of ACN theoretical plate number per meter (N/m) was higher than 10,000, in other systems they were lower. In systems containing methanol in all tested mobile phases (concentration of methanol from 40% to 90%) symmetrical peaks were observed. In systems with methanol higher N/m values for vortioxetine were also obtained, compared to those obtained in systems with acetonitrile. The application of mixture of acetonitrile and methanol in mobile phase lets to obtain symmetrical peaks in almost all used concentrations of these solvents (Table S2). N/m values obtained in the mobile phase with mixed organic modifier were similar or higher compared with those obtained in systems with only methanol or acetonitrile.

The improvement of system efficiency was obtained then vortioxetine was analyzed on Hydro RP column using mobile phase containing mixture of acetonitrile, acetate buffer at pH 3.5 and 0.025 M DEA (Table S3). N/m values were in range between 18,300 in mobile phase containing 50% acetonitrile to 56,170 in mobile phase containing 25% of acetonitrile. However, in the mobile phase system, asymmetrical peaks for vortioxetine were obtained in the whole range of acetonitrile concentrations (As values were from 1.77 to 2.19). The peak symmetry only slightly improved in mobile phase with the addition of diethylamine in which methanol was used as the organic modifier (1.66 < As > 1.97). Systems efficiency were similar to obtained with mobile phases containing acetonitrile. The influence of mobile phases containing various concentration of mixed organic modifier (methanol and acetonitrile) on retention, symmetry of peaks and systems efficiency were also examined (Table S4). The application of mobile phase systems with mixed modifier did not improve the shape of vortoxetine peak. High N/m values were obtained in systems containing 20% or 25% of methanol and acetonitrile each, but in the mobile phases vortioxetine was strongly retained.

#### 2.1.2. Analysis of Vortioxetine on Phenyl Stationary Phase

The next experiments were carried out on Polar RP column with phenyl stationary phase. After application of mobile phase containing acetonitrile, water and formic acid, only for concentrations between 65% to 80% of acetonitrile, symmetrical peaks for vortioxetine were observed. However, N/m values obtained in this range of acetonitrile concentrations were only from 8070 to 10,870 (Table S5). 

Mobile phases containing various concentrations of methanol and 0.1% of formic acid were also examined (Table S5). The concentration 80% of methanol was most optimal for analysis of vortioxetine. In the system symmetrical peak and over 28,000 of N/m was obtained.

Using mixed organic modifier on Polar RP column in six concentration symmetrical peaks for investigated drug were obtained, but N/m values were only from 6630 to 24,430 (Table S6). 

The improvement of peak shape and systems efficiency was obtained on Polar RP column with mobile phases containing addition of DEA (Table S7). In systems with methanol in all ranges of examined methanol concentrations (25%–89%) symmetrical peaks for vortioxetine were observed. High N/m values were also obtained in the whole range of methanol concentrations (from 47,750 in mobile phase containing 70% of methanol to 82,050 in mobile phase containing 25% of methanol) (Table S7). 

The application of mobile phase with mixture of methanol and acetonitrile allowed to obtain symmetrical peaks and high systems efficiency in the whole range of modifier concentrations (Table S8). Obtained vortioxetine As values were from 1.16 in the mobile phase containing 35% methanol, 35% acetonitrile, 20% acetate buffer at pH 3.5 and 0.025 M DEA to 1.21 obtained in mobile phases containing 25% methanol, 25% acetonitrile or, 50% methanol, 10% acetonitrile, 20% acetate buffer at pH 3.5 and 0.025 M DEA. N/m values obtained in mobile phases with mixture of organic modifiers and addition of DEA were from 43,690 to 73,660. Application of chromatographic systems with double protection (phenyl stationary phase with π–π interaction and mobile phase with addition of DEA as free silanol blocker) against undesirable interactions of the basic compound, which was vortioxetine, with free silanol groups lets to obtaining of high system efficiency and symmetrical peaks compared to these obtained on octadecyl stationary phase without π–π interaction.

#### 2.1.3. Analysis of Vortioxetine by HILIC 

Vortioxetine was also analyzed on three different HILIC columns: ACE HILIC-A with silica stationary phase, ACE HILIC-B with aminopropyl phase and ACE HILIC-N with polyhydroxy stationary phase (Table 1). HILIC is suitable for the analysis of polar compounds including polar neutral and polar ionized analytes such as basic compounds. The mechanism of HILIC retention is complicated and involves combinations of hydrophilic interactions, ion exchange, and interactions typical for reversed-phase. Two mobile phases containing 90% ACN and formate buffer at pH 3.8 or 90% ACN, water and 0.1 M ammonium formate were applied on all HILIC columns. Vortioxetine was weakly retained on HILIC B and HILIC N columns with application of both mobile phases (t_R_ between 3.06 to 3.49 min). Stronger retention was observed on HILIC A column especially with mobile phase containing 90% ACN, water and 0.1 M ammonium formate (t_R_ = 6.26 min). Very symmetrical peaks of vortioxetine were obtained in all examined HILIC systems (0.91 < As > 1.17). 

Significantly higher systems efficiency on HILIC A and HILIC B columns were obtained with mobile phase containing 90%ACN and formate buffer at pH 3.8 (N/m values were 394,510 and 183,320, respectively) compared to obtained with mobile phase containing 90% ACN, water and 0.1 M ammonium formate (N/m= 10,290 and 88,800, respectively), while on HILIC N column similar N/m values were obtained with both mobile phases (N/m = 136,170 and 153,230). Based on the experiments in HILIC mode, it can be concluded that most optimal chromatographic parameters for analysis of vortioxetine was obtained on HILIC-A column with mobile phase containing formate buffer (t_R_ = 4.888, As = 1.01 and N/m = 394,510).

#### 2.1.4. Analysis of Vortioxetine by Ion-Exchange Chromatography

An alternative approach to modulate the retention and separation of polar, ionizable compounds such as vortioxetine is ion exchange chromatography (IEC). The retention of compounds in this method depends primarily on the choice of stationary phases, the ionic strength of eluent (type and concentration of buffer), pH and, in some cases, the addition of organic modifiers. In our experiments vortioxetine was analyzed on SCX column with mobile phases containing 25% ACN and phosphate buffer at pH 2.5 or formate buffer at pH 4.0. The psychotropic drug was strongly retained on the SCX column when the mobile phase with addition of formate buffer was applied (t_R_ = 6.931 min) (Table 1). In both mobile phases symmetrical peaks were observed (As = 1.15 and 1.02 in systems with phosphate and formate buffer, respectively). N/m values obtained for vortioxetine by IEC were 15,810 in mobile phase with phosphate buffer and 32,260 in mobile phase containing formate buffer. The results indicate that on SCX column application of mobile phase containing acetonitrile and formate buffer compared to mobile phase with phosphate buffer is more suitable for analysis of vortioxetine.

### 2.2. Determination of the Lipophilicity of Vortioxetine

HPLC is also a powerful technique for measurement of physicochemical properties, e.g., parameters of lipophilicity such as a partition coefficient. The lipophilic character of a compound is the main parameter responsible for its affinity for biological membranes and determine the transport inside an organism, as well as its biopartitioning and bioconcentration. The lipophilicity of substance may be expressed by the logarithm of the partition coefficient obtained from the determination of the compounds distribution between an immiscible polar and nonpolar solvents. However, the measurement of partition coefficients by equilibration methods is often difficult, therefore, it can be determined from chromatographic data [15]. The retention factor is related to the solute distribution between the mobile and stationary phases what is similar to those of water organic solvent systems. In chromatographic methods of partition coefficient determination, most often, retention factor is extrapolated to 100% water (log *kw*). For determination of log *kw* of vortioxetine various chromatographic systems were applied (Table 2A). In most applied mobile phases similar values of log *kw* were obtained (about 4.3), except log *kw* values obtained in systems containing MeCN, acetic buffer at pH 3.5 + 0.025 M DEA and MeOH, acetic buffer at pH 3.6 + 0.01 M octane-1-sulfonic acid sodium salt (OSA-Na) (kog *kw* were 6.52 and 6.10, respectively). For calculation of Log *P* values computer programs can be also used. A number of different computer programs have been developed for the calculation of lipophilicity of chemical compounds based on their structure. In this work, seven computer programs based on different calculation methods for computing log *P* were compared (Table 2B). Similar values of log *P* were calculated by various programs (between 3.86 to 4.92). Average log *P* values calculated by used programs was 4.44 and was similar to the average log *kw* value obtained by HPLC (4.3).

### 2.3. Optimization of Sample Pre-Treatment Method

Sample pre-treatment procedure was appropriate optimized to select the most optimal conditions. The developed SPE method let to obtain good sample purification, satisfactory vortioxetne recovery, and good detection sensitivity. The extraction recovery, extraction efficiency and matrix effects obtained for vortioxetine in various biological samples are presented in Table 3. 

For saliva collection Salivette system was applied. The application of Salivette system in combination with optimal SPE procedure allowed for good results for vortioxetine isolation from saliva matrix (Table 3). 

### 2.4. Analysis of Vortioxetine in Serum, Urine, and Saliva Samples

The optimal extraction and chromatographic procedures were applied to analyze of vortioxetine in serum, saliva and urine samples from patient. The analyses were performed by HPLC-DAD and HPLC-Q-TOF-MS. 

#### 2.4.1. Analysis of Vortioxetine in Serum, Saliva, and Urine Samples by HPLC-DAD

**Figure 1:**
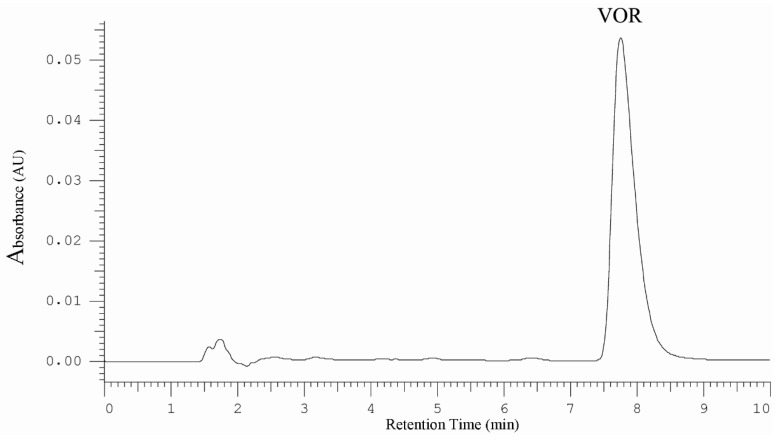
HPLC-DAD chromatogram obtained for vortioxetine standard at concentration 20 µg/mL.

The application of Polar RP column with phenyl moieties and mobile phase with addition of DEA as free silanol blocker allowed to obtaining symmetrical peak, high system efficiency and separation of vortioxetine peak from matrix components. Figure 1 presents typical HPLC-DAD chromatogram obtained for vortioxetine standard at concentration 20 µg/mL. 

The same chromatographic system was successfully used for the analysis of vortioxetine in serum, saliva and urine samples. In all investigated samples vortioxetine and its some metabolites were identified (Table 4). The vortioxetine peak was fully separated from matrix components. The peak purity indexes for vortioxetine peaks were greater than 0.99 in all cases. The identities of the analyte peaks in biological samples were confirmed by the comparison of their retention times, UV-Vis spectra with those obtained for vortioxetine standard.

**Figure 2:**
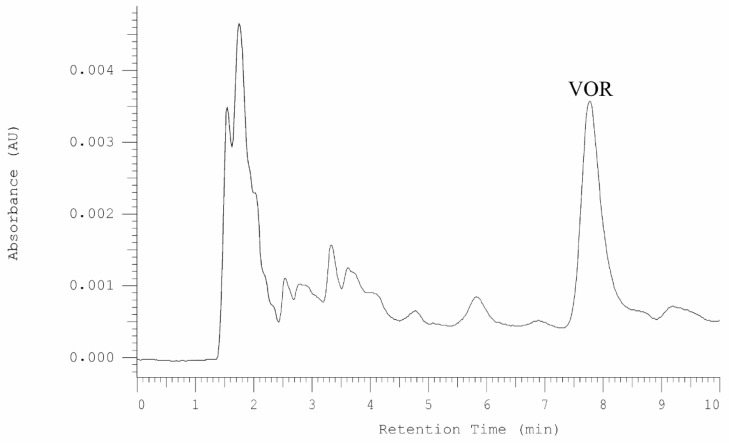
HPLC-DAD chromatogram obtained for serum sample from patent treated by vortioxetine. The sample was taken 24 h after drug administration.

**Figure 3:**
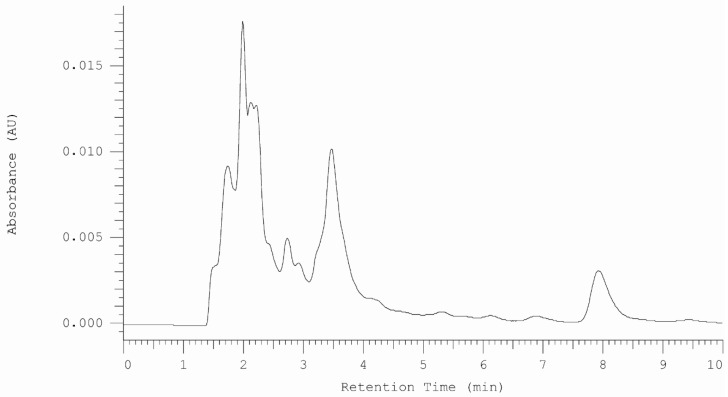
HPLC-DAD chromatogram obtained for urine sample from patent treated by vortioxetine. The sample was taken 24 h after drug administration.

Serum and urine samples were taken from patient after 24 h after drug administration. In both samples vortioxetine was detected (Figure 2 and Figure 3).

**Figure 4:**
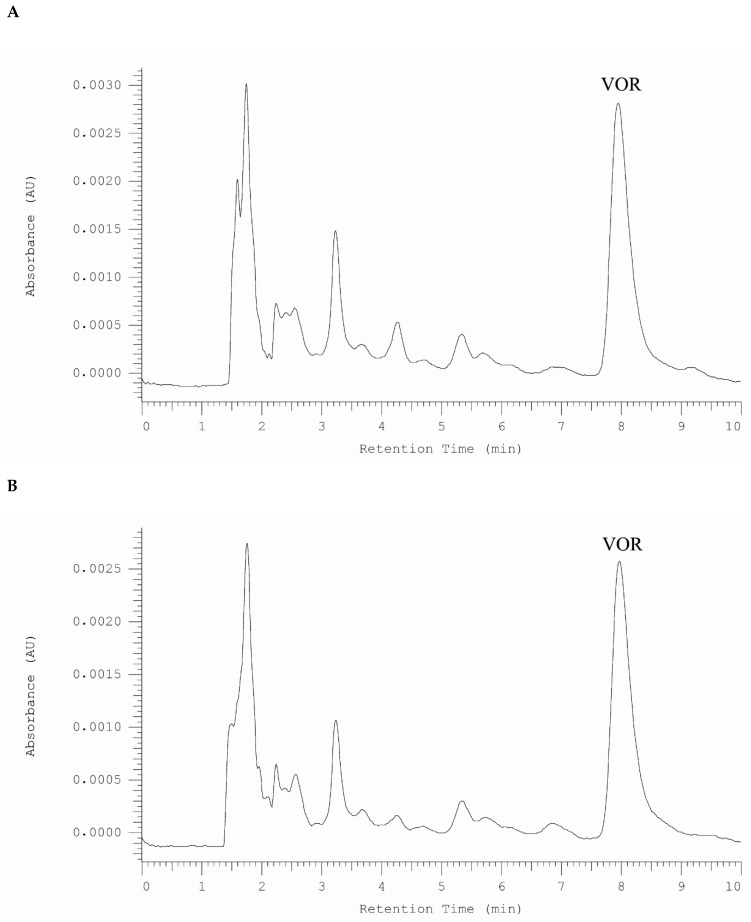
HPLC-DAD chromatogram obtained for saliva sample from patient treated by vortioxetine. The sample was taken (**A**). 1 h and (**B**). 24 h after drug administration.

For the first time, vortioxetine was detected in patient’s saliva. The saliva samples were collected at 1 h, 2 h, 4 h, 8 h, 10 h, and 24 h after drug administration. In all saliva samples vortioxetine was determined. Examples of chromatograms obtained after 1 h and 24 h after drug administration are presented in Figure 4.

#### 2.4.2. Analysis of Vortioxetine in Serum, Saliva and Urine Samples by LC-QTOF-MS

**Figure 5:**
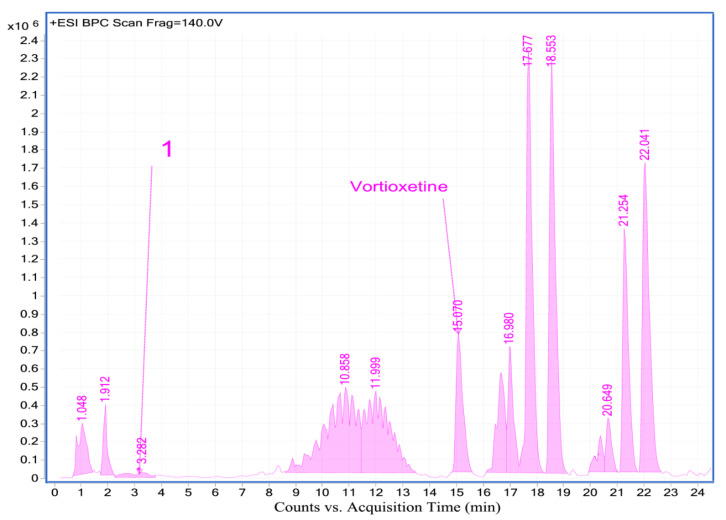
LC-MS chromatogram obtained for serum sample from patient treated with vortioxetine.

**Figure 6:**
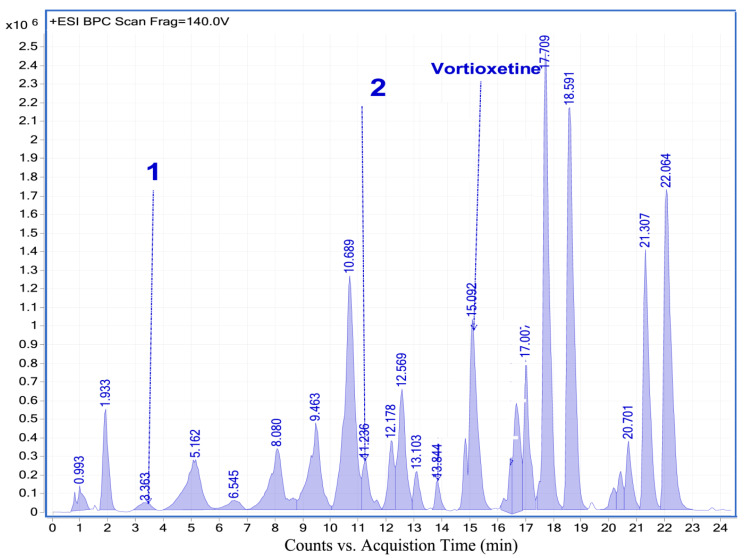
LC-MS chromatogram obtained for urine sample from patient treated with vortioxetine.

**Figure 7:**
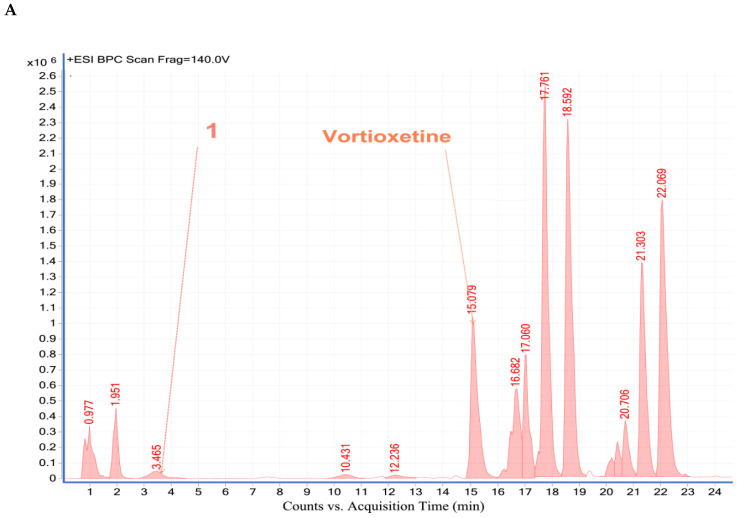

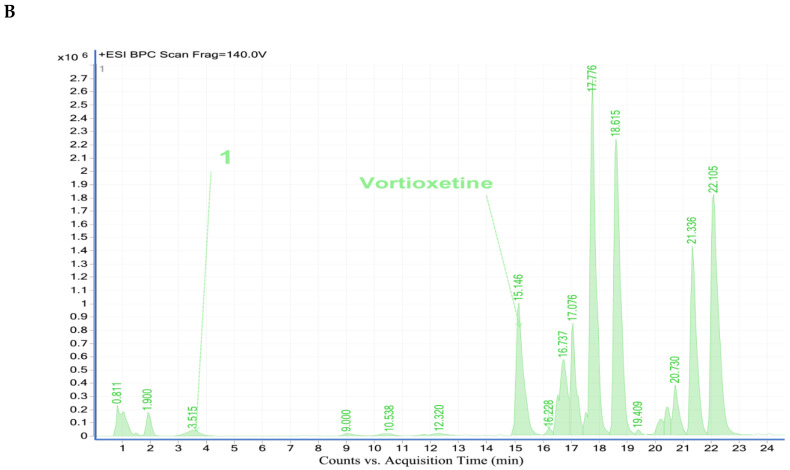
LC-MS chromatogram obtained for saliva sample from patient treated with vortioxetine. The sample was taken (**A**). 1 h and (**B**). 24 h after drug administration.

For additional confirmation of vortioxetine presence in biological samples LC-QTOF-MS analysis was applied. Retention time, formula, molecular ion and fragment ion for vortioxetine and its some metabolites are presented in Table 5. In investigated samples from patient two metabolites: LU AE22404 and LU AA34443 have been detected. LC-MS chromatograms obtained for biological samples are presented in Figure 5, Figure 6 and Figure 7. Exemplary extracted ion chromatograms for vortioxetine and its metabolites detected in samples from patient are presented as supplementary materials (Figures S1–S9). Serum and urine samples were collected 24 h after vortioxetine administration. In serum sample vortioxetine and metabolite LU AE22404 were detected. In the urine sample obtained from the same patient at the same time after drug administration presence of vortioxetine and metabolites LU AE22404, LU AA34443 were confirmed. 

Presence of vortioxetine and its metabolites in saliva samples from patient collected from 1 h to 24 h after vortioxetine administration were also confirmed by MS spectra. In all samples vortioxetine and matabolite LU AE22404 were determined for the first time. The obtained results indicate the possibility of application of saliva samples to determination of vortioxetine concentrations in patients, e.g., for therapeutic drug monitoring. The use of saliva compared to serum or plasma for detection of psychotropic drugs has been shown to be an attractive alternative for therapeutic drug monitoring in psychiatric patients because of its collection are simpler, non-invasive, and painless. The interesting is also determination of vortioxetine in urine samples because in the sample more metabolites have been identified that in serum or saliva.

## 3. Materials and Methods 

### 3.1. Chemicals and Reagents

Standard of vortioxetine (purity = 99.8%) was obtained from H. Lundbeck A/S (Copenhagen, Denmark). Methanol (MeOH), acetonitrile (ACN) of chromatographic quality, diethylamine (DEA), octane-1-sulfonic acid sodium salt (OSA-Na) for HPLC, acetic acid (99%–100%), formic acid (98%–100%), sodium acetate, ammonium formate, ammonium (25%), ammonium chloride, and water for LC-MS were purchased from Merck (Darmstadt, Germany). Water for LC-DAD analysis was double distilled. 

### 3.2. Apparatus and LC Conditions

The LC-DAD and LC-QTOF-MS analyzes were performed using various chromatographic systems. The LC conditions are described below. The efficiency of chromatographic systems was calculated as theoretical plate number expressed as N/m (per meter of column) and peak symmetry as asymmetry factor (As).

#### 3.2.1. LC-DAD Conditions

The chromatographic analyzes were performed using various chromatographic columns which parameters are presented in Table 6. Detection of vortioxetine in biological samples was conducted using Synergi Polar RP column. The analyzes were performed at 22 °C with an eluent flow rate of 1.0 mL/min. Samples (30 μL) were injected onto the HPLC column and analyzed in isocratic mode with eluent consisting of methanol 32% *v/v*, acetonitrile 28% *v/v*, acetate buffer (pH 3.5) 20% *v/v*, double distilled water 20% *v/v*, and 0.025 M L^−1^ diethylamine. The DAD detector was set in the 200–400 nm range, and qualitative analysis was performed at 250 nm. Retention time for vortioxetine was 7.87 min.

Vortioxetine was detected in all investigated biological samples. Peak purity was confirmed by comparison of UV spectra obtained for investigated drug in biological samples with the spectra of standard. Peak purity index for drug’s peaks was found to be greater than 0.99.

#### 3.2.2. LC-QTOF-MS Conditions

The samples were also analyzed qualitatively by HPLC/ESI-QTOF-MS system in positive ion mode using a 630B accurate mass QTOF-MS (Agilent Technologies INc., Santa Clara, CA, USA) mass spectrometer with an ESI-Jet-Stream ion source. The chromatograph was equipped with binary gradient pump, autosampler, column oven (20 °C) and DAD detector. Separation of analytes was performed on Gemini 3 μm C18 110Ǻ (100 × 2.1 mm) column. Water with 0.1% HCOOH was used as mobile phase A and acetonitrile with 0.1% HCOOH as a mobile phase B. The following gradient was applied: 0–45 min 15%–80% B; 45–46 min 80%–95% B; 46–50 min 95% B; post time 10 min. Flow rate was 0.3 mL/min, injection volume was 10 µL, total time of analysis was 60 min. ESI-QTOF-MS analysis was performed with following parameters. Dual spray Jet Stream, positive ion mode, gas (N_2_) temperature: 300 °C, flow rate 12 mL/min, 35 psig, sheath gas temperature: 325 °C, flow rate 12 L/min, 35 psig, fragmentor voltage 140 V, Vcap 4000 V. Collision induced cell at two energies 10 and 40 eV. Data acquisition was performed in Auto MS/MS mode at the range of 100–1000 mass units for MS and MS/MS. Mass Hunter B.07.00 software (Agilent Technologies INc., Santa Clara, CA, USA) was used for data analysis.

### 3.3. Serum, Saliva, and Urine Samples Collection 

Serum, saliva and urine samples were obtained from healthy volunteers (for SPE procedure optimization) or from patient (clinical application). For clinical application samples were collected from patient-man, 51 years old, BMI = 29, non-smoker, virtually no alcohol drinker, somatically healthy, does not take any medicine except vortioxetine 10 mg/day. Patient has been suffering from depression since 2010, burdened with bipolar disorder. The study was conducted on the 15th day of taking vortioxetine. 

The study protocol was approved by the Bioethical Committee of the Medical University of Lublin (approval number KE-0254/254/2017).

#### 3.3.1. Serum 

Blood sample was collected from patient at steady-state 24 h after the last dose (10 mg). After blood coagulation the samples were centrifuged for 10 min at 1500× *g*. The serum was separated and stored at −20 °C until analysis.

#### 3.3.2. Saliva

Saliva samples were collected from patient 1, 2, 4, 8, 10, and 24 h after the last dose (10 mg). Salivette (Sarstedt, Nümbrech, Germany) was used for sample collection. The patient removed the swab from the Salivette and places the swab in the mouth and chews it for about 60 s to stimulate salivation. Next the swab with the absorbed saliva was returned to the salivette. Then salivette was centrifuged for 2 min at 1000× *g* yields to obtain clear saliva sample in the conical tube. The saliva was stored at −20 °C until analysis.

#### 3.3.3. Urine

Urine was collected to sterile container in the morning from healthy volunteers or patient 24 h after dose of vortioxetine (10 mg). The urine was stored at −20 °C until analysis.

### 3.4. SPE Procedure for Isolation of Vortioxetine from Serum, Saliva, and Urine

Solid phase extraction (SPE) procedure was carried out using BAKERBOND^TM^spe Octadecyl J.T. Baker cartridge, 100 mg/mL) on a Baker spe-12G apparatus. 

#### 3.4.1. Preparation of Serum Samples 

The serum samples were diluted with 1mL of ammonium buffer at pH 8.6. The extraction column was activated with 1 mL of methanol and conditioned with 1 mL of a mixture containing water and ammonium buffer at pH 8.6 (5:1 *v/v*). Then, the serum was introduced to the SPE column at a speed 1 mL/min. The column was prewashed with 1 mL of MeOH–water solution (1:1 *v/v*) and dried under vacuum for 3 min. The extracted drug was eluted twice with 1 mL of mixture containing 98% methanol and 2% acetic acid (*v/v*). The sample was evaporated to dryness and dissolved in 0.32 mL of MeOH. Then aliquot of the eluate was injected directly into the HPLC column. 

#### 3.4.2. Preparation of Saliva Samples 

The saliva samples were diluted with 0.2 mL of ammonium buffer at pH 8.6. The SPE extraction column was activated with 1 mL of methanol and conditioned with 1 mL of a mixture containing water and ammonium buffer at pH 8.6 (5:1 *v/v*). Next, the previously prepared saliva samples were introduced to the column at a speed of 1 mL/min. Then, the column was prewashed with 1 mL of MeOH–water solution (1:1 *v/v*) and dried applying vacuum for 3 min. The extracted drug was eluted twice with 1 mL of mixture containing 98% methanol and 2% acetic acid (*v/v*). The sample was evaporated to dryness and dissolved in 0.32 mL of MeOH. The aliquot of the eluate was injected directly into the HPLC column.

#### 3.4.3. Preparation of Urine Samples 

The urine samples were diluted with 1mL of ammonium buffer at pH 8.6. The extraction column was activated with 1 mL of methanol and conditioned with 1 mL of a mixture containing water and ammonium buffer at pH 8.6 (5:1 *v/v*). Then, the urine was introduced to the SPE column at a speed 1 mL/min. The column was prewashed with 1 mL of MeOH–water solution (1:1 *v/v*) and dried under vacuum for 3 min. The extracted drug was eluted twice with 1 mL of mixture containing 98% methanol and 2% acetic acid (*v/v*). The sample was evaporated to dryness and dissolved in 0.32 mL of MeOH. Next, aliquot of the eluate was injected directly into the HPLC column.

### 3.5. Matrix Effects, Process Efficiency, and Extraction Recovery 

Extraction recovery, extraction efficiency, and matrix effects were calculated by equations:

Recovery (%) = C/B * 100

Extraction Efficiency (%) = C/A * 100

Matrix effect (%) = B/A * 100

Were:

A = external solution peak area, B = post-extraction sample peak area, C = extracted matrix peak area.

### 3.6. Preparation of Stock Solution and Working Solutions

The stock standard solution of vortioxetine was prepared in methanol at a concentration of 1 mg/mL by dissolving an amount of vortioxetine hydrobromide corresponding to 50 mg of free base in 50 mL methanol and stored at −20 °C, protected from light. The working standard solutions of vortioxetine were prepared by diluting of the above mentioned stock solution in methanol before analysis.

### 3.7. Log P Calculation

#### 3.7.1. Calculation by Computer Programs 

In this study, seven software based on different calculation methods for computing Log *P*: miLogP (http://www.molinspiration.com), ALOGPS (http://146.107.217.178/servlets/vcclab?action=alogps), ChemAxon (https://chemaxon.com/products/calculators-and-predictors), ALogP http://146.107.217.178/servlets/vcclab?action=alogps), CLOGP (http://www.biobyte.com/bb/prod/clogp40.html), XLogP3-AA (https://www.scribd.com/document/122098618/XLOGP3), CLogP (http://www.biobyte.com/bb/prod/clogp40.html) were applied.

#### 3.7.2. Determination by LC

For determination of log *kw* of vortioxetine various HPLC systems were applied. The analyses were performed using Hydro RP or Polar RP columns and different composition of mobile phases (Table 2A).

## 4. Conclusions

Great differences in retention, peak shapes and systems efficiency of vortioxetine and its metabolites were obtained various chromatographic systems: RP with alkylbonded or phenyl stationary phases, HILIC and IEC). The highest N/m values and very symmetrical peak for vortioxetine was obtained on HILIC a column with mobile phase containing 90% ACN and formate buffer pH 3.8. The good shape peak and high system efficiency were also obtained on SCX column with mobile phase containing formate buffer and on Polar RP column with mobile phase containing addition of DEA.

Selected chromatographic systems were applied for determination by extrapolation of vortioxetine log *kw* value. Obtained log *kw* were good correlated with whose obtained by calculation methods.

In the current study, the HPLC method was developed for determination of vortioxetine and its metabolites in human serum, urine, and saliva samples obtained for patient treated with the drug. 

The applied SPE process allowed for effectively remove interfering substances from the matrix and optimal HPLC procedure permitted for the analysis of vortioxetine and its metabolites in serum, urine, and saliva samples obtained from psychiatric patient. All investigated samples—serum, urine, and saliva collected from patient treated with vortioxetine can be used for the drug determination, because in all samples vortoxetine and its metabolites were easily detected.

For the first time, vortioxetine was detected in patient’s saliva. Determination of vortioxetine in saliva samples is an attractive alternative for determination of the drug for therapeutic drug monitoring in psychiatric patients because of its collection is non-invasive and pain-free. An important advantage of using saliva samples for the determination of vortioxetine is also that the drug and its metabolites were detected as soon as 1 h after the administration of the drug and it was also possible to detect them 24 h after its administration, immediately before the next dose.

For the first time, a comparison of a large number of different chromatographic systems (RP, HILIC, ICE) for the detection of vortioxetine was performed. The obtained results can be used for the development of analytical methods for qualitative and quantitative analysis of vortioxetine in various types of samples by HPLC coupled with different detection techniques.

For the first time, lipophilicity parameters for vortioxetine were determined by LC using various chromatographic systems and were compared with values calculated using computer programs.

## Figures and Tables

**Table 1:** Values of t_R_, log k, A_S_, and N/m obtained for vortioxetine in various chromatographic systems.

Stationary Phase	Mobile Phase	t_R_	As	N/m
HILIC A	90% ACN and formate buffer pH 3.8	4.89	1.01	394,510
HILIC A	90% ACN 0.1 M HCOONH_4_	6.26	0.91	10,290
HILIC B	90% ACN and formate buffer pH 3.8	3.06	1.17	183,320
HILIC B	90% ACN 0.1 M HCOONH_4_	3.10	1.01	88,800
HILIC N	90% ACN and formate buffer pH 3.8	3.49	1.08	136,170
HILIC N	90% ACN 0.1 M HCOONH_4_	3.45	1.01	153,230
SCX	25% ACN, and formate buffer 2.5	3.15	1.15	15,810
SCX	25% ACN and formate buffer pH 3.8	6.93	1.02	32,260
Hydro RP	80% MeOH, acetate buffer at pH 3.6 0.01 M OSNa	4.74	0.77	13,170
Hydro RP	75% MeOH acetate buffer at pH 3.6 0.01 M OSNa	8.72	0.80	19,370
Hydro RP	70% MeOH acetate buffer at H 3.6 0.01 M OSNa	17.41	0.80	27,540
Hydro RP	65% MeOH acetate buffer at pH 3.6 0.01 M OSNa	39.40	0.89	36,800
Hydro RP	60% MeOH acetate buffer at pH 3.6 0.01 M OSNa	90.92	0.95	51,910
Polar RP	80% MeOH acetate buffer at pH 3.6 0.01 M OSNa	3.66	1.12	18,890
Polar RP	75% MeOH acetate buffer at pH 3.6 0.01 M OSNa	5.00	0.96	18,060
Polar RP	70% MeOH acetate buffer at pH 3.6 0.01 M OSNa	7.34	0.92	19,190
Polar RP	65% MeOH acetate buffer at pH 3.6 0.01 M OSNa	12.59	0.91	22,290
Polar RP	60% MeOH acetate buffer at pH 3.6 0.01 M OSNa	25.80	0.94	27,890
Polar RP	55% MeOH acetate buffer at pH 3.6 0.01 M OSNa	59.32	1.01	36,550

**(A):** 

Chromatographic System		Log *kw*
Column	Mobile Phase	
Hydro RP	MeCN + 0.1% HCOOH	4.06
	MeOH + 0.1% HCOOH	4.73
	MeOH, acetic buffer at pH 3.5 + 0.025 M DEA	4.00
	MeCN, acetic buffer at pH 3.5 + 0.025 M DEA	6.52
	MeOH, acetic buffer at pH 3.6 + 0.01 M OSA-Na	6.10
Polar RP	MeOH, acetic buffer at pH 3.5 + 0.025 M DEA	4.25
	MeCN, acetic buffer at pH 3.5 + 0.025 M DEA	4.23
	MeOH b.oct. pH 3.6 0.01 M OSA-Na	4.65
	Average	4.82

**(B):** 

Program	Log *P*
miLogP	4.08
ALOGPS	4.51
ChemAxon	4.76
ALogP	3.86
CLOGP	4.92
XLogP3-AA	4.20
cLogP	4.76
Average	4.44

**Table 3:** Recovery, extraction efficiency and matrix effect obtained for vortioxetine.

Sample	Concentration Added (ng/mL)	Recovery (%)	Extraction Efficiency (%)	Matrix Effect (%)
Serum	40	105.26	111.05	105.51
Urine	40	76.11	81.41	106.96
Saliva	40	101.93	75.62	74.19

**Table 4:** Chemical structure of vortioxetine and its metabolites.

Compound	Structure
Vortioxetine (Lu AA21004)	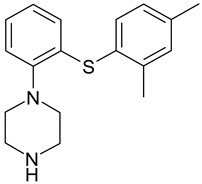
Lu AA34442 (M0)	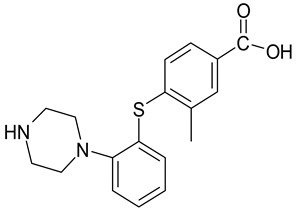
Lu AE22404	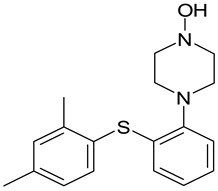

**Table 5:** Retention time, formula, molecular ion, fragment ion for vortioxetine and its metabolites, biological samples where vortioxetine/metabolites were detected.

No	Tentatively Assignment	Retention Time [min]	Formula	Molecular Ion [M+H]^+^	Fragment Ions	Biological Samples Where Vortioxetine/ Metabolite was Detected							
						Serum	Saliva						Urine
							1 h	2 h	4 h	8 h	10 h	24 h	
	Vortioxetine	15.09	C_18_H_23_N_2_S^+^	299.1557	150.0341; 109.0068	+	+	+	+	+	+	+	+
1	LU AE22404	3.36	C_18_H_23_N_2_OS^+^	315.1539	191.0625; 120.0780	+	+	+	+	+	+	+	+
2	LU AA34443 (M0)	11.24	C_18_H_23_N_2_O_2_S^+^	329.1366	286.0877; 150.0367; 109.0088	-	-	-	-	-	-	-	+

+ detected, - not detected.

**Table 6:** List of tested columns and their physicochemical properties.

Column	Functional Group	Lenght (mm)	I.D. (mm)	Endcaped	Particle Size (μm)	Pore Size (Å)	Surface Area (m2/g)	Carbon Load (%)
Synergi Polar RP	Ether-linked phenyl	150	4.6	Proprietary (polar group)	4	80	475	11
Synergi Hydro-RP	Octadecyl (C18)	150	4.6	Proprietary (polar group)	4	80	475	19
ACE HILIC-A	Proprietary SIL	150	4.6	NO	5	100	300	-
ACE HILIC-B	Proprietary Aminopropyl	150	4.6	NO	5	100	300	4
ACE HILIC-C	Proprietary Polyhydroxy	150	4.6	NO	5	100	300	7
Luna SCX	Benzene Sulfonic Acid	150	4.6	NO	5	100	400	0.55 Sulfur Load

## Supplementary Materials

The following are available online at https://www.mdpi.com/1420-3049/25/11/2483/s1.
