# Supplementary material for: Comparison of Various Chromatographic Systems for Identification of Vortioxetine in Bulk Drug Substance, Human Serum, Saliva, and Urine Samples by HPLC-DAD and LC-QTOF-MS
Source: Molecules. 2020 May 27;25(11):2483. doi: 10.3390/molecules25112483 (PMC7321162; doi:10.3390/molecules25112483)
Supplement: Supplementary file 1 [file molecules-25-02483-s001.pdf]

Supplementary materials

Table S1. A. Values of t_R, log k, A_S, and N/m obtained for vortioxetine on Hydro PR column.

ACN, water, 0.1% HCOOH				
%ACN	t_R [min]	log k	As	N/m
30	26.1	1.32	3.45	12480
40	5.42	0.56	2.85	8380
50	3.65	0.32	2.3	7180
60	2.51	0.05	2.15	8000
70	2.21	-0.06	1.97	7610
80	1.65	-0.4	*	3220
90	2.08	-0.12	*	2570

Table S1B. Values of t_R, log k, A_S, and N/m obtained for vortioxetine on Hydro PR column.

MeOH, water, 0.1% HCOOH				
%MeOH	t_R [min]	log k	As	N/m
40	87.97	2.01	1.16	28090
50	25.8	1.46	1.2	8770
60	6.74	0.82	1.03	16260
70	2.15	0.06	1.08	10430
80	1.42	-0.5	0.97	4230
90	1.22	-1.3	1.16	11510

* very asymmetrical peak

Table S2. Values of t_R, log k, A_S, and N/m obtained for vortioxetine on Hydro PR column.

MeOH, ACN, water, 0.1% HCOOH					
%MeOH	%ACN	t_R [min]	log k	As	N/m
17.5	17.5	40.66	1.52	1.39	24703
20	20	22.19	1.25	2.17	47080
25	25	5.59	0.57	*	27343
30	30	2.53	0.06	1.25	17163
35	35	1.38	-0.77	*	2623
40	40	1.33	-0.9	1.08	4937
50	10	3.7	0.33	1.09	13370
40	20	3.03	0.2	1.21	15797
20	40	1.98	-0.17	0.94	16673

10	50	1.25	-1.26	1.36	1367

* very asymmetrical peak

Table S3A. Values of t_R, log k, A_S, and N/m obtained for vortioxetine on Hydro PR column.

ACN, water, 20% acetate buffer at pH 3.5, 0.025M DEA				
%ACN	t_R [min]	log k	As	N/m
25	162	2.14	2.19	56170
30	31.5	1.41	1.82	49920
40	6.94	0.69	1.88	25630
50	3.79	0.34	1.77	18300
60	3.65	0.32	1.82	18390
70	4.39	0.43	1.83	20630
80	5.36	0.55	1.88	23640

Table S3B. Values of t_R, log k, A_S, and N/m obtained for vortioxetine on Hydro PR column.

MeOH, water, 20% acetate buffer at pH 3.5, 0.025M DEA				
%MeOH	t_R [min]	log k	As	N/m
50	75.27	1.94	1.66	22700
55	38.7	1.65	1.73	27660
60	22.13	1.39	1.97	32470
65	12.98	1.14	1.87	29500
70	8.53	0.94	1.87	26950
80	4.29	0.56	1.66	23990

Table S4. Values of t_R, log k, A_S, and N/m obtained for vortioxetine on Hydro PR column.

MeOH, ACN, water, acetate buffer at pH 3.5, 0.025M DEA					
%MeOH	%ACN	t_R [min]	log k	As	N/m
20	20	89.75	1.88	1.92	60650
25	25	30.81	1.4	1.81	72810
30	30	12.3	0.97	1.78	45820
35	35	6.03	0.61	1.64	36700
40	40	4.04	0.38	1.61	32470
50	10	19.42	1.19	1.76	13370
40	20	15.15	1.07	1.8	15800
20	40	8.16	0.77	1.96	16670

10	50	5.69	0.58	1.94	1370

Table S5A. Values of t_R, log k, A_S, and N/m obtained for vortioxetine on Polar PR column.

ACN, water, 0.1% HCOOH				
%ACN	t_R [min]	log k	As	N/m
20	101.25	2.01	2.37	20880
30	11.59	1.06	2.35	14870
40	2.61	0.42	2.26	13010
50	1.04	0.02	1.81	12720
55	0.76	-0.12	1.62	12610
60	0.64	-0.19	1.54	12190
65	0.54	-0.27	1.4	10870
70	0.53	-0.27	1.33	9260
75	0.61	-0.22	1.3	8070
80	0.79	-0.10	1.45	9740
85	1.30	0.11	1.6	19500
90	2.87	0.46	1.63	31280
95	9.06	0.96	1.48	3750

Table S5B. Values of t_R, log k, A_S, and N/m obtained for vortioxetine on Polar PR column.

MeOH, water, 0.1% HCOOH				
%MeOH	t_R [min]	log k	As	N/m
40	74.76	74.76	1.61	22650
50	16.33	16.33	1.99	14920
55	7.91	7.91	1.83	20360
60	4.22	4.22	1.95	15060
70	1.28	1.28	1.61	16850
80	0.35	0.35	1.37	28350
90	*			

* very asymmetrical peak

Table S6. Values of t_R, log k, A_S, and N/m obtained for vortioxetine on Polar PR column.

MeOH, ACN, water, 0.1% HCOOH					
%MeOH	%ACN	t_R [min]	log k	As	N/m
15	15	103	1.79	1.86	24430
20	20	23	1.11	1.99	18490
25	25	7.7	0.57	1.84	16490

30	30	4.09	0.17	1.51	17710
35	35	2.85	-0.14	1.49	22300
40	40	1.65	-	1.02	6630
50	10	6.22	0.44	1.26	16690
40	20	4.75	0.27	1.35	16650
20	40	3.41	0.03	1.44	19460
10	50	2.72	-0.19	1.20	19460

Table S7. Values of t_R, log k, A_S, and N/m obtained for vortioxetine on Polar PR column.

ACN, water, 20% acetate buffer at pH 3.5, 0.025M DEA					MeOH, water, 20% acetate buffer at pH 3.5, 0.025M DEA				
%ACN	t_R [min]	log k	As	N/m	%MeOH	t_R [min]	log k	As	N/m
25	92.53	1.71	1.35	82050	40	221.5	2.12	0.97	45380
30	35.69	1.29	1.33	79320	50	54.19	1.5	1.03	20940
40	10.47	0.7	1.28	65970	60	15.83	0.93	1.19	27110
50	5.51	0.33	1.24	59250	70	7.03	0.51	1.25	33750
60	4.06	0.12	1.19	52460	80	4.12	0.18	1.22	33890
70	3.62	0.03	1.22	47750					
80	3.92	0.09	1.23	48560					

Table S8. Values of t_R, log k, A_S, and N/m obtained for vortioxetine on Polar PR column.

MeOH, ACN, water, acetate buffer at pH 3.5, 0.025M DEA					
%MeOH	%ACN	t_R [min]	log k	As	N/m
20	20	43.65	1.41	1.20	73660
25	25	15.16	0.91	1.21	57640
30	30	7.04	0.51	1.17	53360
35	35	4.47	0.23	1.16	48060
40	40	3.45	0.04	1.18	45050
10	50	13.21	0.85	1.19	43690
20	40	9.30	0.67	1.19	49970
40	20	5.90	0.41	1.18	55040
50	10	4.81	0.28	1.21	54510

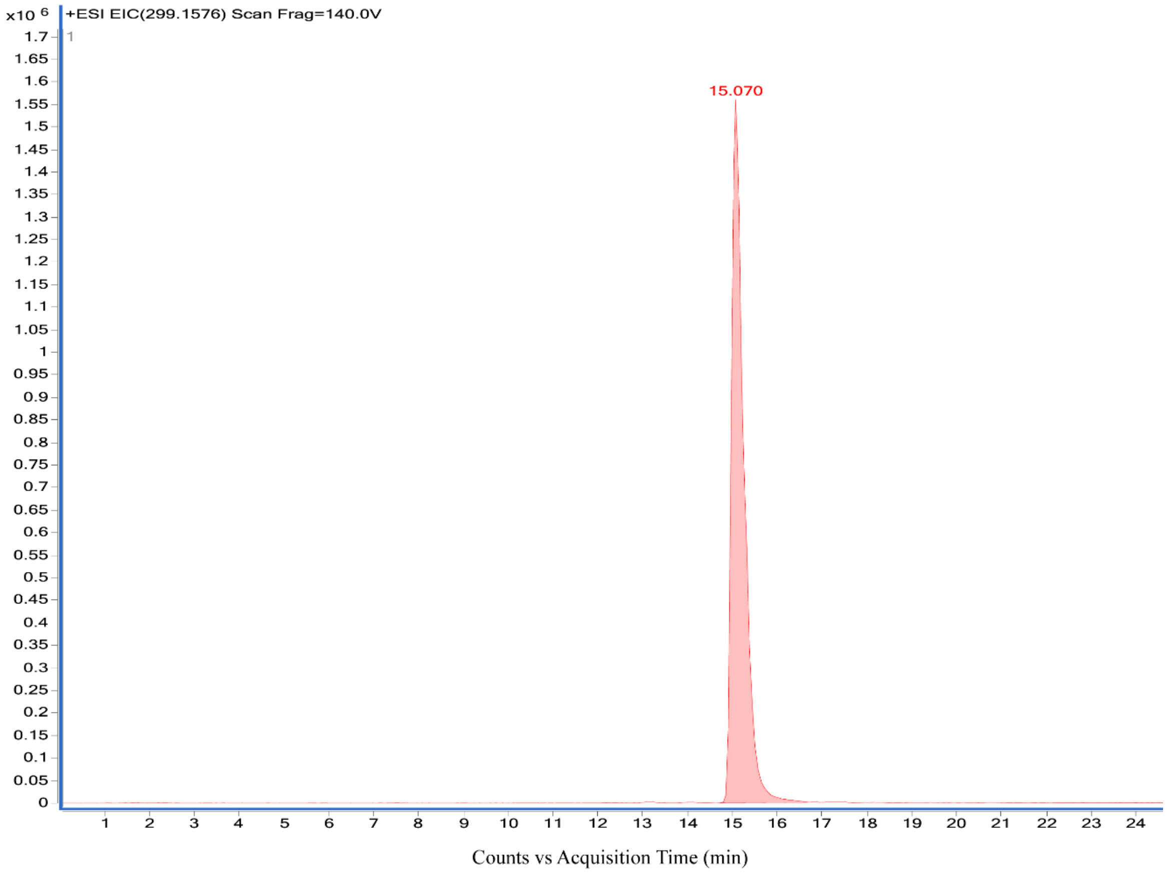

Figure S1. Extracted ion chromatogram obtained for vortioxetine in serum sample. The sample was taken 24h after drug administration

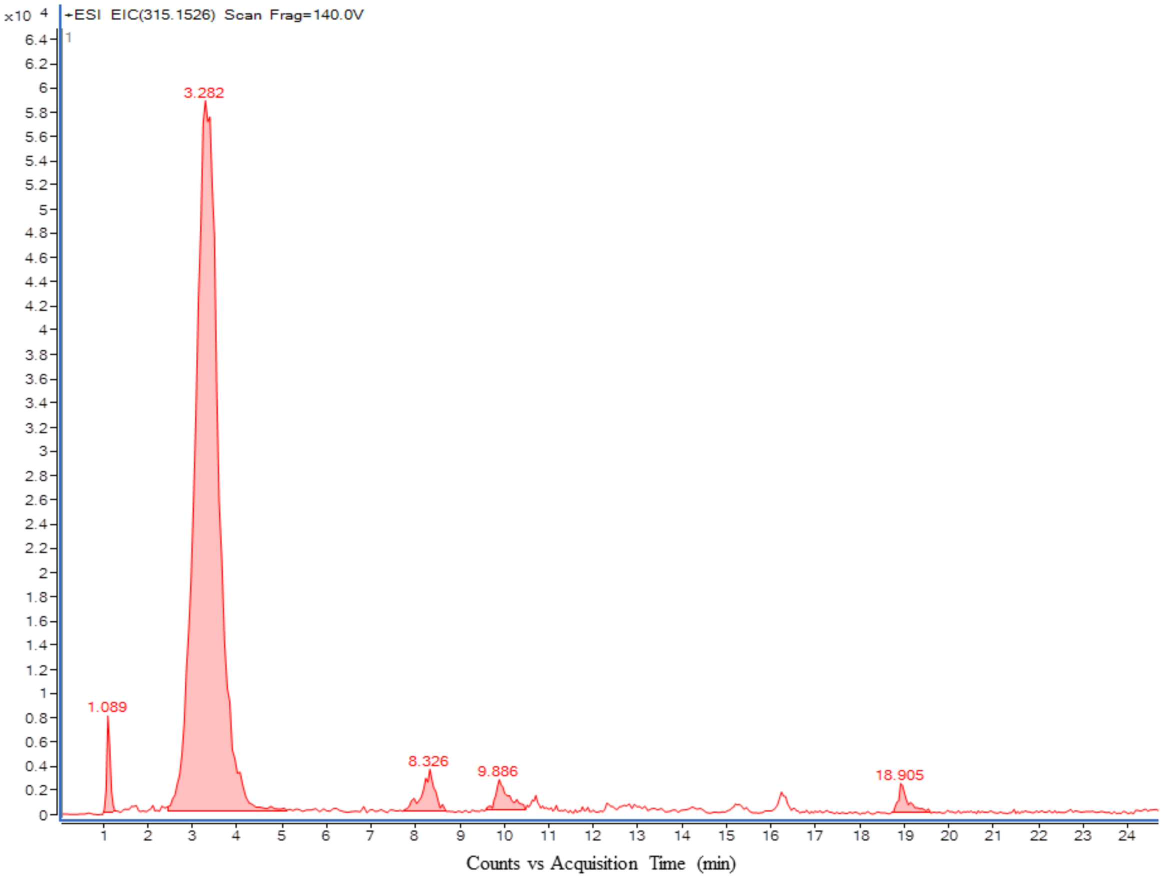

Figure S2. Extracted ion chromatogram obtained for metabolite LU AE22404 in serum sample. The sample was taken 24h after drug administration.

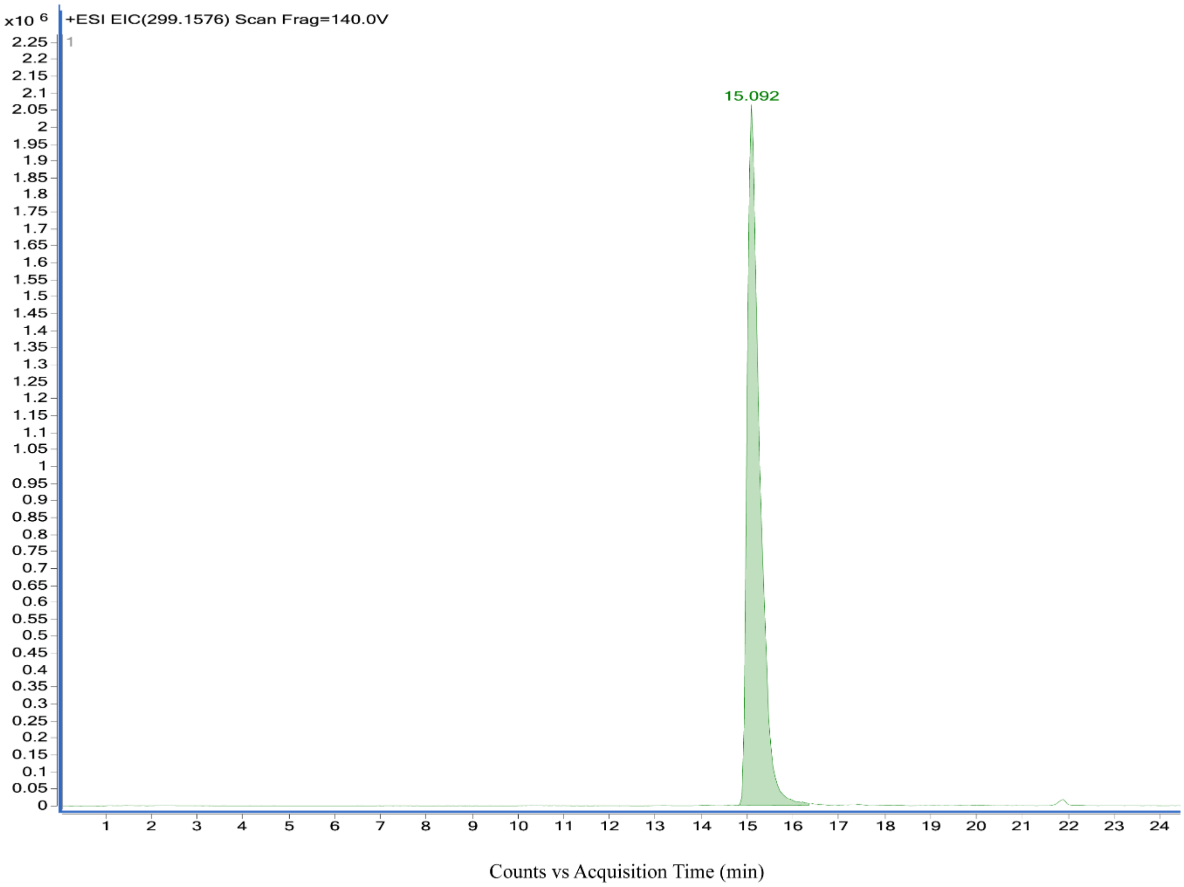

Figure S3. Extracted ion chromatogram obtained for vortioxetine in urine sample. The sample was taken 24h after drug administration.

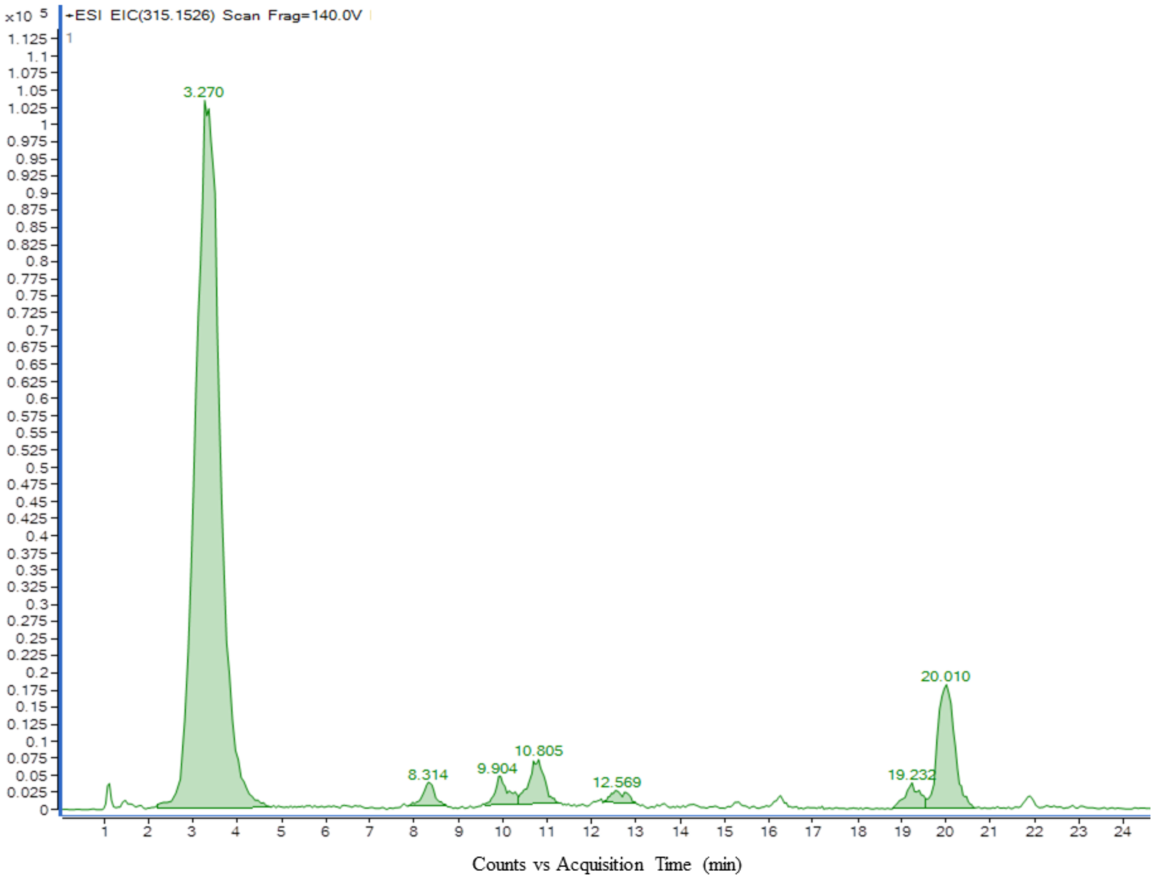

Figure S4. Extracted ion chromatogram obtained for metabolite LU AE22404 in urine sample. The sample was taken 24h after drug administration.

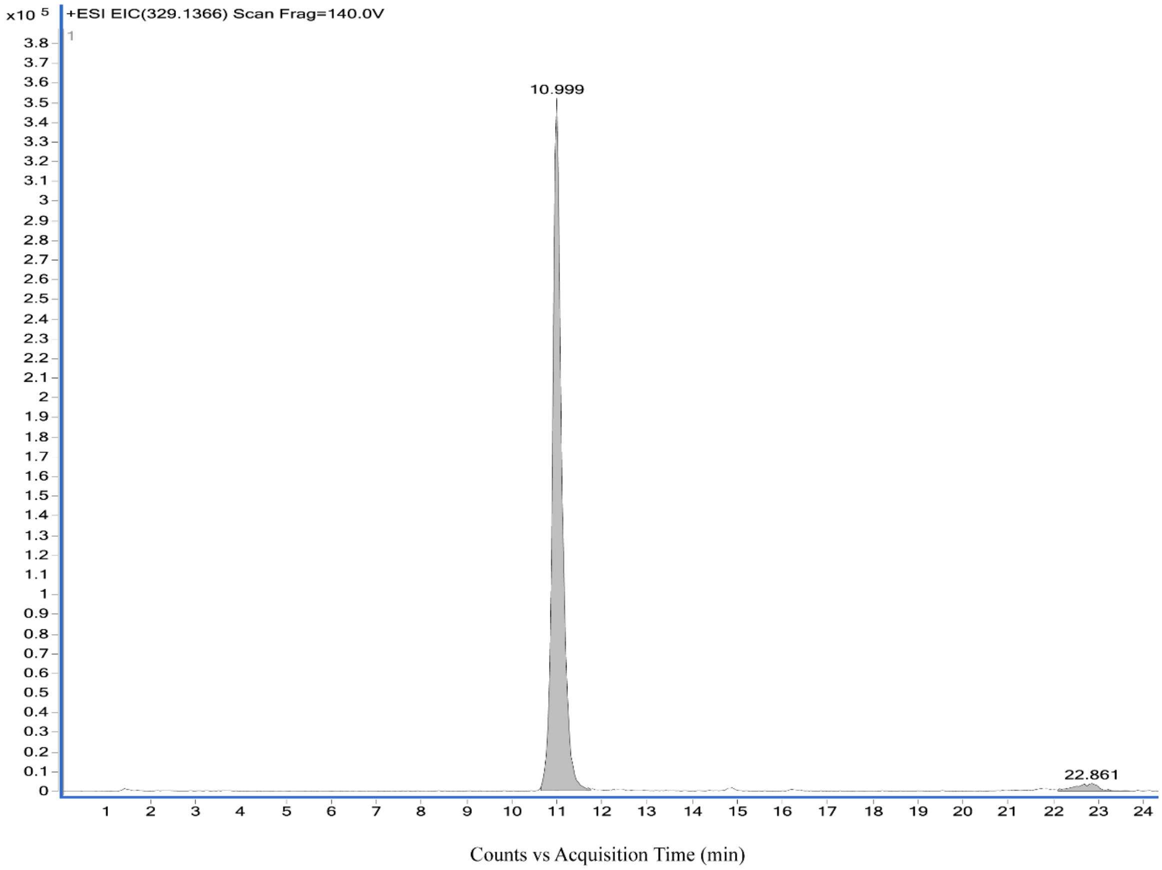

Figure S5. Extracted ion chromatogram obtained for metabolite LU AA34443 in urine sample. The sample was taken 24h after drug administration.

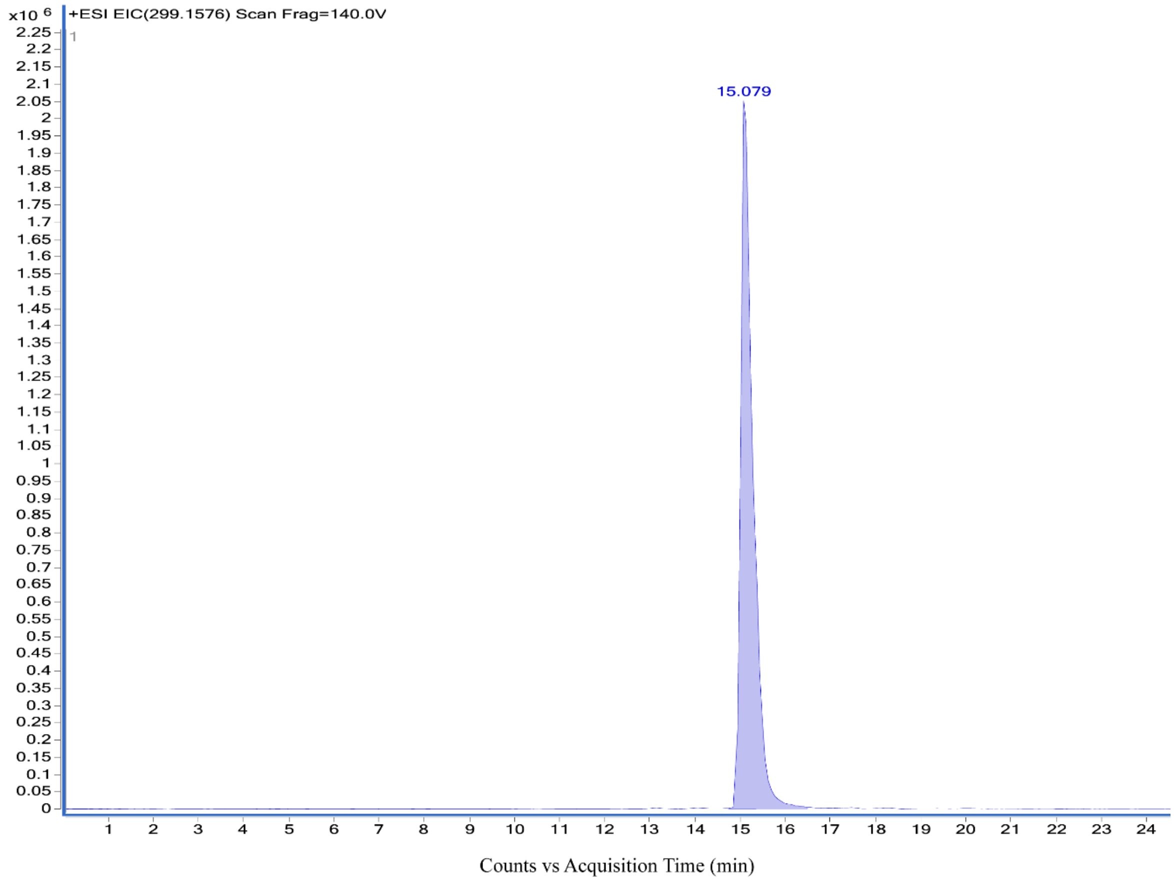

Figure S6. Extracted ion chromatogram obtained for vortioxetine in saliva sample. The sample was taken 1h after drug administration.

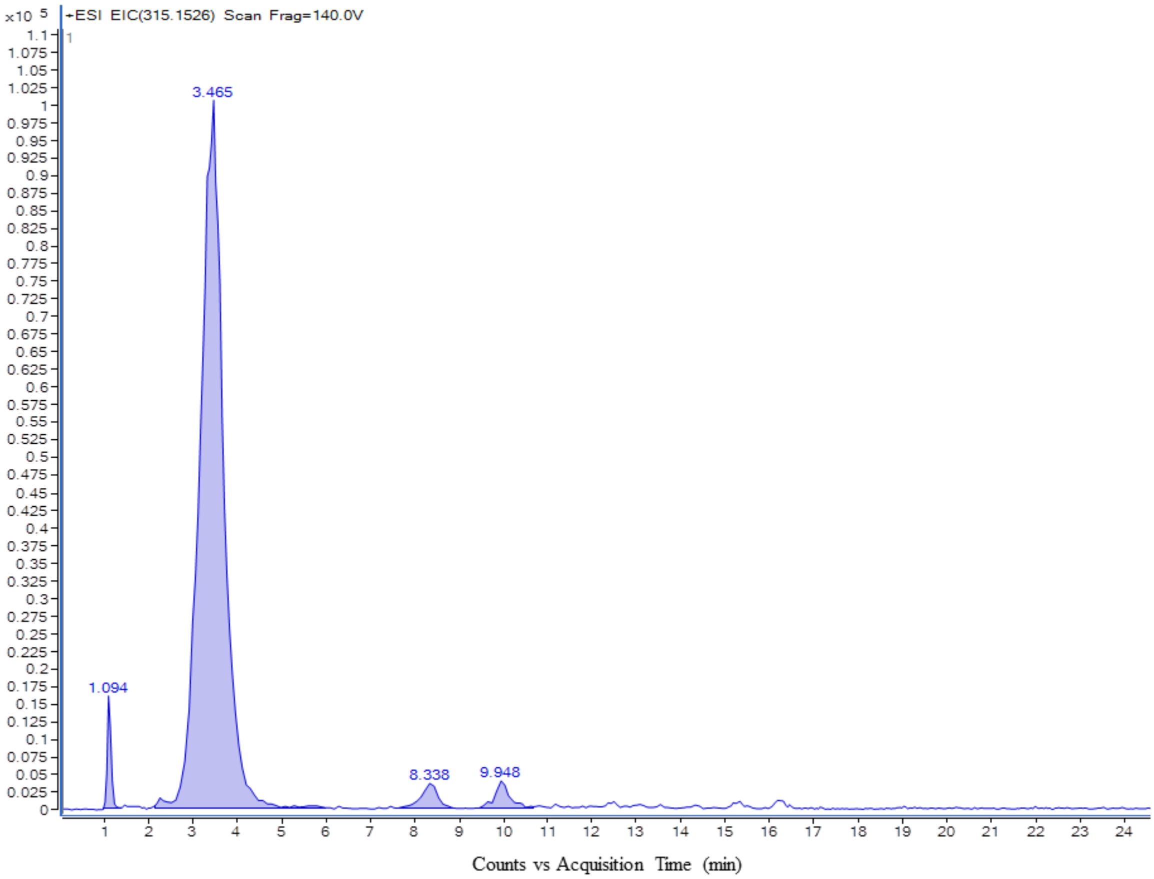

Figure S7. Extracted ion chromatogram obtained for metabolite LU AE22404 in saliva sample. The sample was taken 1h after drug administration.

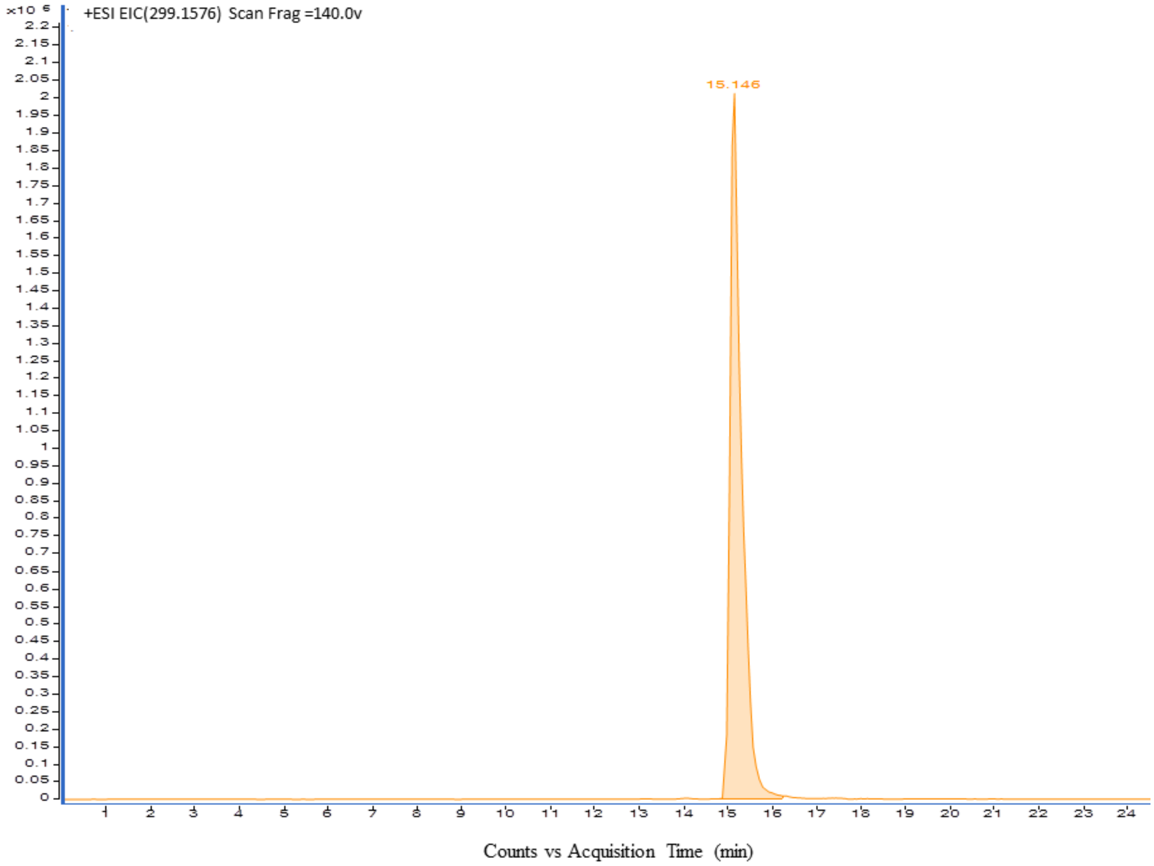

Figure S8. Extracted ion chromatogram obtained for vortioxetine in saliva sample. The sample was taken 24h after drug administration.

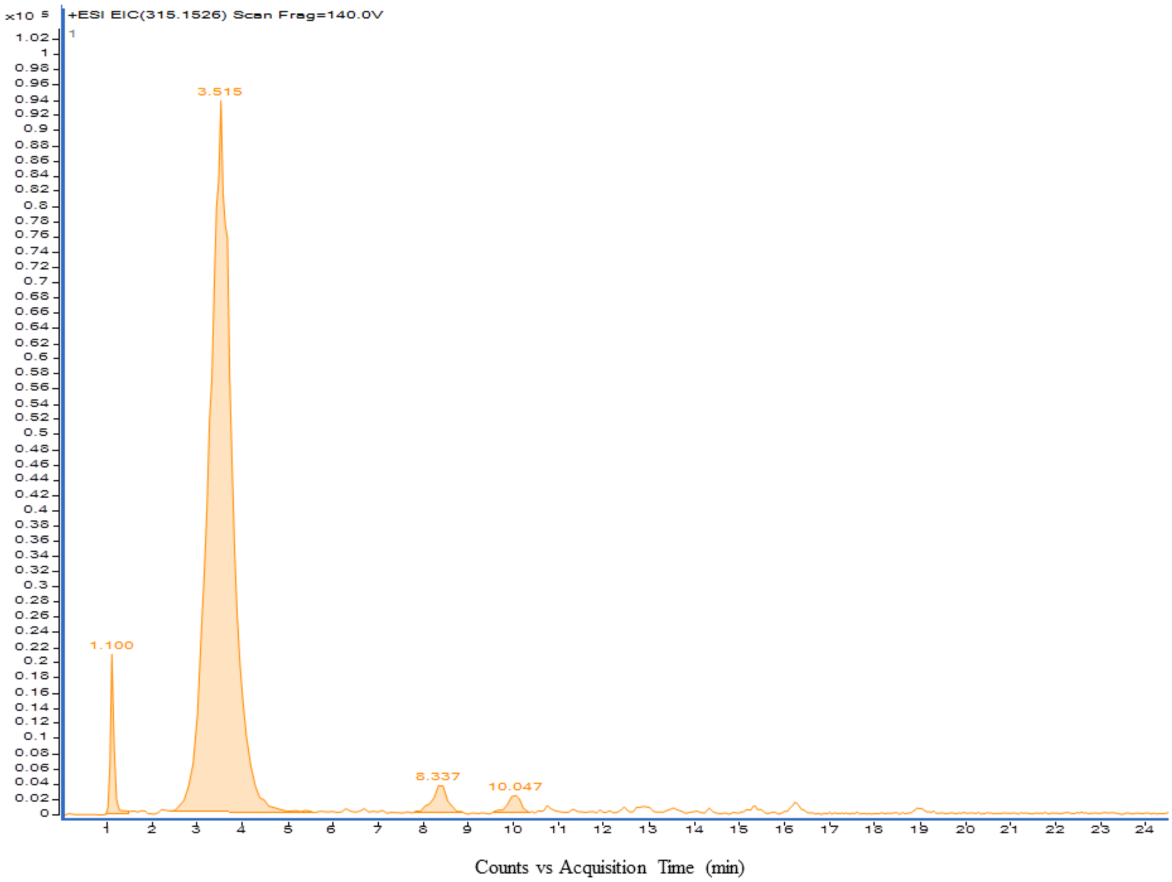

Figure S9. Extracted ion chromatogram obtained for vortioxetine in saliva sample. The sample was taken 24h after drug administration.
